# The Development of a Dual-Warhead Impact System for Dynamic Linearity Measurement of a High*-g* Micro-Electro-Mechanical-Systems (MEMS) Accelerometer

**DOI:** 10.3390/s16060840

**Published:** 2016-06-08

**Authors:** Yunbo Shi, Zhicai Yang, Zongmin Ma, Huiliang Cao, Zhiwei Kou, Dan Zhi, Yanxiang Chen, Hengzhen Feng, Jun Liu

**Affiliations:** 1 Science and Technology on Electronic Test and Measurement Laboratory, North University of China, Taiyuan 030000, Shanxi, China; shiyunbo@nuc.edu.cn (Y.S.); yangzhicai@nuc.edu.cn (Z.Y.); caohuiliang@nuc.edu.cn (H.C.); kouzhiwei@imut.edu.cn (Z.K.); nuc_zhidan@163.com (D.Z.); chenyanxiang_nuc@163.com (Y.C.); fenghengzhen@nuc.edu.cn (H.F.); 2 Key Laboratory of Instrumentation Science & Dynamic Measurement, Ministry of Education, North University of China, Taiyuan 030000, Shanxi, China

**Keywords:** dual-warhead Hopkinson bar, laser interferometer, dynamic linearity, accelerometer, measurement

## Abstract

Despite its extreme significance, dynamic linearity measurement for high*-g* accelerometers has not been discussed experimentally in previous research. In this study, we developed a novel method using a dual-warhead Hopkinson bar to measure the dynamic linearity of a high*-g* acceleration sensor with a laser interference impact experiment. First, we theoretically determined that dynamic linearity is a performance indicator that can be used to assess the quality merits of high*-g* accelerometers and is the basis of the frequency response. We also found that the dynamic linearity of the dual-warhead Hopkinson bar without an accelerometer is 2.5% experimentally. Further, we verify that dynamic linearity of the accelerometer is 3.88% after calibrating the Hopkinson bar with the accelerometer. The results confirm the reliability and feasibility of measuring dynamic linearity for high*-g* accelerometers using this method.

## 1. Introduction

High*-g* accelerometers are one of the most important inertial devices [1,2,3,4] and have been widely applied to measuring dynamic impact and high-speed motion during overload, especially in the field of inertia measurement and automatic control [5,6,7]. Calibrations in amplitude and dynamic sensitivity for accelerometers have been reported by Bateman *et al.* and Lee *et al.* [8,9,10,11]. Bateman *et al*. suggested that the key factors affecting the dynamic performance of accelerometers are amplitude linearity and frequency characteristics [8,9,10], and they also experimentally discussed performance characteristics including time-domain amplitude linearity, which reflects the relationship between the sensitivity of the sensor, the acceleration amplitude, and frequency-domain characteristics of a model 7280 A Endevco accelerometer. The calibration data shown in their papers matched frequency- and time-domain requirements. The frequency-domain requirement imposed a frequency response that was flat to within ±5% across the frequency range of interest, and the time domain requirement is that, if the sensitivity is based upon the low amplitude vibration calibration, it is critical that the linearity characteristics of the shock-based “Amplitude Linearity” be understood such that an amplitude measurement uncertainty is clearly defined [9,11,12,13,14]. However, they did not realize the importance of dynamic linearity for an accelerometer, which is rarely researched but is especially important for accelerometer calibration [15,16,17,18]. In [13], Akira Umeda described the dynamic linearity as being “used to represent the relationship between the sensor input value, output value, and frequency, which reflects the relationship between the sensor range and the working frequency band.” In accordance with the linear superposition principle of a one-dimensional stress wave, Umeda put forward the principle of the dynamic linear test [13]. In this study, for the first time, a novel method for calibrating the dynamic linearity of an accelerometer based on a dual-warhead Hopkinson bar was developed. Firstly, we theoretically determined the dynamic linearity with the output of the accelerometer. We then setup the dual-warhead Hopkinson bar experiment to experimentally confirm these findings and conclude that the dynamic linearity of the dual-warhead Hopkinson bar is 2.5% without a high*-g* piezoresistive accelerometer. Based on the experiment, the dynamic linearity of the high*-g* piezoresistive accelerometer was found to be 3.88%.

## 2. The Principle of Dynamic Linearity Measurement

Firstly, it is known that a linear system has the property listed below:

If the inputs are *X*_1_(*t*) and *X*_2_(*t*) and the corresponding outputs are recorded as *Y*_1_(*t*) and *Y*_2_(*t*), respectively, then, when the input is *aX*_1_(*t*) + *bX*_2_(*t*), the output is *Y*_1+2_(*t*), namely, *aY*_1_(*t*) + *bY*_2_(*t*), where a and b are constants. 

In fact, considering the errors of the system, the dynamic linearity of the system σ is given by the following equation:

(1) $$\sigma =\frac{\left|{aY}_{1}(t)+{bY}_{2}(t)-Y_{1+2}(t)\right|}{Y_{1+2}(t)}\times 100\%$$

**Figure 1:**
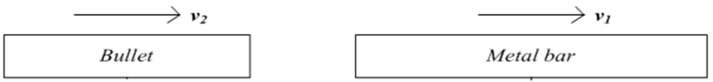
Coaxial impact of a limited elastic bar by a bullet and a metal bar.

It is believed that the one-dimensional stress wave theory can be used under the condition that the propagation pulse wavelength is at least 6–10 times longer than that of the diameter of a bar, shown in Figure 1 [19]. The velocity of the stress wave propagation in Hopkinson bar is given by the following equation [20,21]:

(2) $$\frac{c}{\sqrt{\frac{E}{\rho }}}\approx 1-r^{2}\pi^{2}{\left(\frac{R}{\lambda }\right)}^{2}$$

where *c*, *E*, *ρ*, *r*, *R*, and *λ* with the values of 5010 m/s, 113 GPa, 4.5 g/cm^3^, 0.3, and 15 mm represent stress wave velocity, elastic modulus, density, Poisson ratio, the bar radius, and the stress wave length, respectively.

The impact acceleration *a*(*t*) is generated at the moment *t* when the reflection of the elastic pulse occurs. One-dimensional stress wave theory can be used to derive the following equation:

(3) $$a(t)=2C\dot{\varepsilon }(t)$$

where *ε*(*t*), *C*, and *a*(*t*) are the strain of an elastic pulse incident upon the end surface, the velocity of the elastic pulse in the bar, and the acceleration of the motion of the end surface of the bar generated during the reflection of the elastic pulse, respectively.

**Figure 2:**
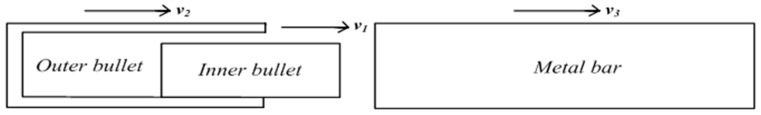
Coaxial impact of the limited elastic bar by the inner and outer bullets and the metal bar.

Figure 2 shows the elastic impact system comprising the inner and outer bullets and the metal bar. The inner bullet is put inside the outer one, and it is assumed that there is no interaction between the bullets except for gravity during coaxial impact. The two bullets can impact the metal bar simultaneously or separately while maintaining the same impact conditions. The incident elastic wave pulse strains of the end surface of the bar are *ε*_*in*,1_(*t*), *ε*_*in*,2_(*t*), and *ε*_*in*,1+2_(*t*) when the inner bullet, outer bullet, and both, respectively, impact the bar. We define the corresponding velocities of the end surface of the bar as *v*_*in*,1_(*t*), *v*_*in*,2_(*t*), and *v*_*in*,1+2_(*t*), based on one-dimensional stress wave theory, and they can be given by the following [19]:

(4) $$\nu_{in,1}(t)=2C\varepsilon_{in,1}(t)$$

(5) $$\nu_{in,2}(t)=2C\varepsilon_{in,2}(t)\;\text{and}$$

(6) $$\nu_{in,1+2}(t)=2C\varepsilon_{in,1+2}(t)$$

Therefore, the corresponding impact accelerations *α*_*in*,1_(*t*), *α*_*in*,2_(*t*), and *α*_*in*,1+2_(*t*), which are the differentials of velocity, at the end of the surface of the bar are given by the following:

(7) $$\alpha_{in,1}(t)={\dot{\nu }}_{in,1}(t)=2C{\dot{\varepsilon }}_{in,1}(t)$$

(8) $$\alpha_{in,2}(t)={\dot{\nu }}_{in,2}(t)=2C{\dot{\varepsilon }}_{in,2}(t)\;\text{and}$$

(9) $$\alpha_{in,1+2}(t)={\dot{\nu }}_{in,1+2}(t)=2C{\dot{\varepsilon }}_{in,1+2}(t)$$

Thus, the dynamic linearity in the elastic impact system of two bullets and a metal bar is as follows:

(10) $$\begin{array}{ll} \sigma & =\frac{\left|(a_{outer}+a_{inner})-a_{total}\right|}{a_{total}}\times 100\%\\ & =\frac{\left|a_{plus}-a_{total}\right|}{a_{total}}\times 100\% \end{array}$$

where *a_outer_*, *a_inner_*, and *a_total_* are the output accelerations for the bar when impacted by the outer bullet, inner bullet, and both, respectively. The *a_plus_* represents the sum of *a_outer_* and *a_inner_*. The *a_total_* represents the dynamic linearity of the impact system.

Consequently, we can obtain the dynamic linearity of the impact system and the accelerometers by directly measuring the accelerations of the bullets. We will discuss how to obtain these results later in this paper.

## 3. Experiment Details

**Figure 3:**
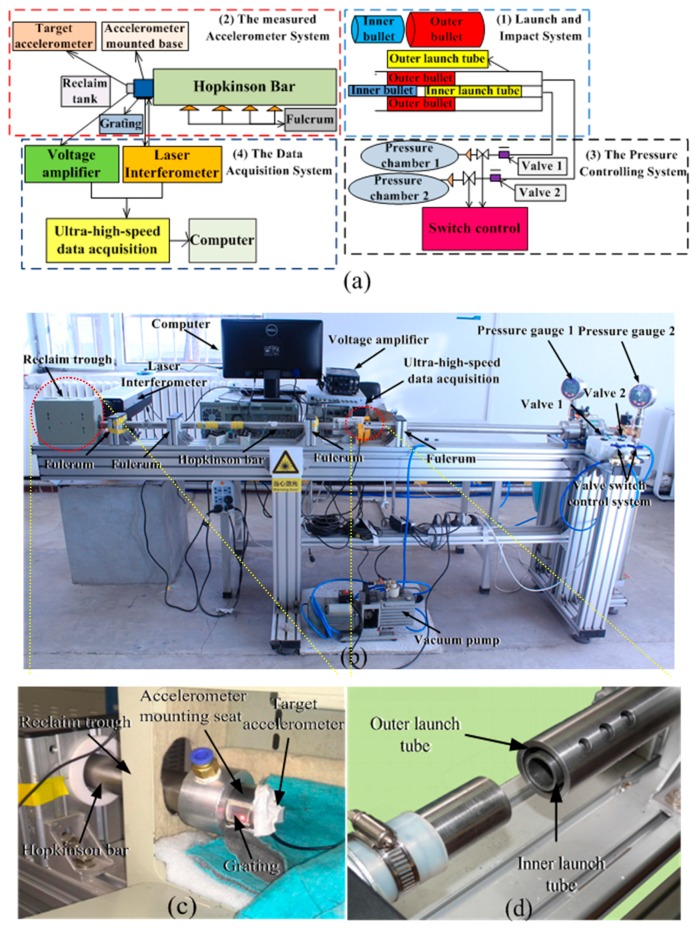
Schematic diagram (**a**) and experimental setup (**b**) for the impact measurement system using a home-made dual-warhead Hopkinson bar for dynamic linearity increment with a high measurement range. (**c**) Close-up of the measured system comprising a target accelerometer, its mounted seat, and grating. (**d**) Close-up of the launch system comprising inner and outer bullets. The outer and inner launch tube and are marked by red circles in (**b**), respectively.

The origin of the use of the Hopkinson bar for characterization of high*-g* accelerometer's performances is worth mentioning. The schematics and experimental setup of the impact system using a developed dual-warhead Hopkinson bar for dynamic linearity increment with a high measurement range are shown in Figure 3.

The impact measurement system can be divided into four subsystems, including a launch and impact system, a measured accelerometer system, the pressure controlling system, and the data acquisition system, as shown in Figure 3a,b. Figure 3c,d show close-ups of the dual-warhead made in-houses, its launch tube and the measured accelerometer marked by both red circles in Figure 3b.

The launch-measured accelerometer and impact systems comprise home-made inner and outer launching tubes for the blast-off of the bullets. The home-made Hopkinson bar fabricated from high-strength titanium is used as the incident bar to generate the strain wave when impacted by the bullets. The inner and outer bullets made of No. 45 quenched and tempered steel are used as the generator of the elastic impact to the Hopkinson bar and are launched by the launching tubes. The parameters of these bullets are shown in Table 1.

The measured accelerometer system comprises a home-made piezoresistive accelerometer [22,23,24], a mounted base, grating, and reclamation tank. The accelerometer is mounted on the end of the base, with a measuring range of 1.5 × 10^5^ *g* and anti-overload reaching of 2.0 × 10^5^ *g*, and is used to measure the acceleration from the Hopkinson bar induced by the impact of the bullets. The grating is mounted beside the base for measuring the base’s Doppler frequency shift due to acceleration. The reclamation tank embraces and recycles the accelerometer, the mounted base, and the grating after the impact.

The pressure control system comprises pressure chambers 1 and 2, valves 1 and 2, and a valve switch. Pressure chambers 1 and 2 are used as high-pressure resources, providing air with a pressure as high as 1 MPa. The switches are used to regulate the air pressure of chambers 1 and 2. Valves 1 and 2 can launch the inner and outer bullets separately or simultaneously to hit the Hopkinson bar.

The data acquisition system comprises a laser interferometer (HSLV-1000, Chang Cheng Institute, China), a voltage amplifier (Endevco 136 with its voltage gain of 100, Endevco, Irvine, CA, USA), and a computer. The laser interferometer converts the Doppler frequency shift from the grating to voltage, which is amplified by the voltage amplifier. The ultra-high-speed USB data acquisition (the acquisition accuracy is 12 bit and the acquisition rate is 40 MHz) and analysis from the amplifier is processed by the computer, yielding results concerning the acceleration and the impact duration.

When performing experiments for which the inner bullet (outer bullet) is launched, pressure chamber 1(2) is opened and regulated by the switch while keeping the air pressure constant at an interval of 0.005 MPa between 0.01 and 0.1 MPa. Then, valve 1(2) is opened, and the inner (outer) tube is pushed to launch the inner (outer) bullet with a high air pressure. The inner (outer) bullet impacts the Hopkinson bar, and a longitudinal elastic compression wave is generated propagating to the other side of the bar. The accelerometer mounted to the side of the bar flies out immediately because of the stretching wave induced by the quasi-half-sine acceleration pulse from the compression wave. The grating, which is mounted to the base, measures the Doppler frequency shift depending of the acceleration of the accelerometer and converts it into a voltage signal, which is finally imported into the OP amplifier and analyzed by the data acquisition system. The acceleration of the inner bullet, *a_inner_* (outer bullet, *a_outer_*) is obtained relative to the impact duration. When both bullets are launched simultaneously under the same condition, pressure chambers 1 and 2, the switches, and the inner and outer tubes are both worked in sequence, and the other procedures are performed just as in the case of one bullet. The acceleration of the inner and outer bullets, *a_total_*, is obtained. The dynamic linearity of the impact system and accelerometer can consequently be calculated depending on Equation (10).

## 4. Experimental Results

In this section, firstly, the dynamic linearity of the impact system without the accelerometer is investigated by measuring the accelerations of the mounted base impacted by the inner bullet, outer bullet, or by both, simultaneously. Using this impact system with high dynamic linearity, the performance of the accelerometer is researched using its recorded outputs under all considered impacts.

### 4.1. The System Calibration Using a Laser Interferometer without an Accelerometer

**Figure 4:**
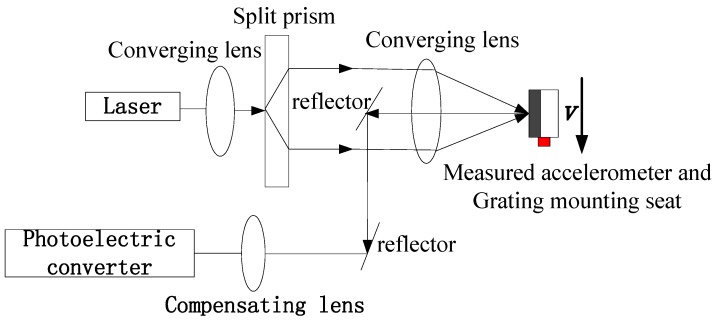
The principle diagram of the differential laser Doppler interferometer.

The acceleration can be obtained using the differential laser Doppler interferometer method which is shown in Figure 4 [25,26,27].

The velocity of the object and the Doppler shift is given by [24,28]

(11) $$v(t)=k_{v}\Delta f(t)$$

where *k_V_* is the velocity sensitivity (*ms*^−1^/*Hz*).

The grating velocity measured by the laser interferometer is given by [29]

(12) $$v(t)=\frac{\lambda }{2sin(i)}\Delta f(t)$$

When the grating is moving, the Doppler frequency shift is given by

(13) $$\frac{\lambda }{sini}=\frac{2d}{\left|m-n\right|}$$

Therefore, substituting Equation (12) into Equation (13), the grating velocity can be given by

(14) $$v(t)=\frac{d}{\left|m-n\right|}\Delta f(t)$$

where *m*, *n* are the diffraction series of two interference beams in the interferometer, and *m* = −*n* = 2. *d*, with a value of 1/150 mm, is the grating constant. Therefore, the velocity sensitivity coefficient of the interferometer is given by

(15) $$k_{v}=\frac{d}{\left|m-n\right|}=1.67\times 10^{-6}\;{ms}^{-1}/Hz$$

Grating acceleration can thus be acquired:

(16) $$a(t)=k_{v}•\frac{d(\Delta f(t))}{dt}$$

For a given grating, when the light path is determined by the diffraction series, we can get the velocity and acceleration of the grating from Δ*f*. Thereby, calibration of the test system using a laser interferometer without an accelerometer is achieved.

### 4.2. Dynamic Linearity Results of the Impact System

**Figure 5:**
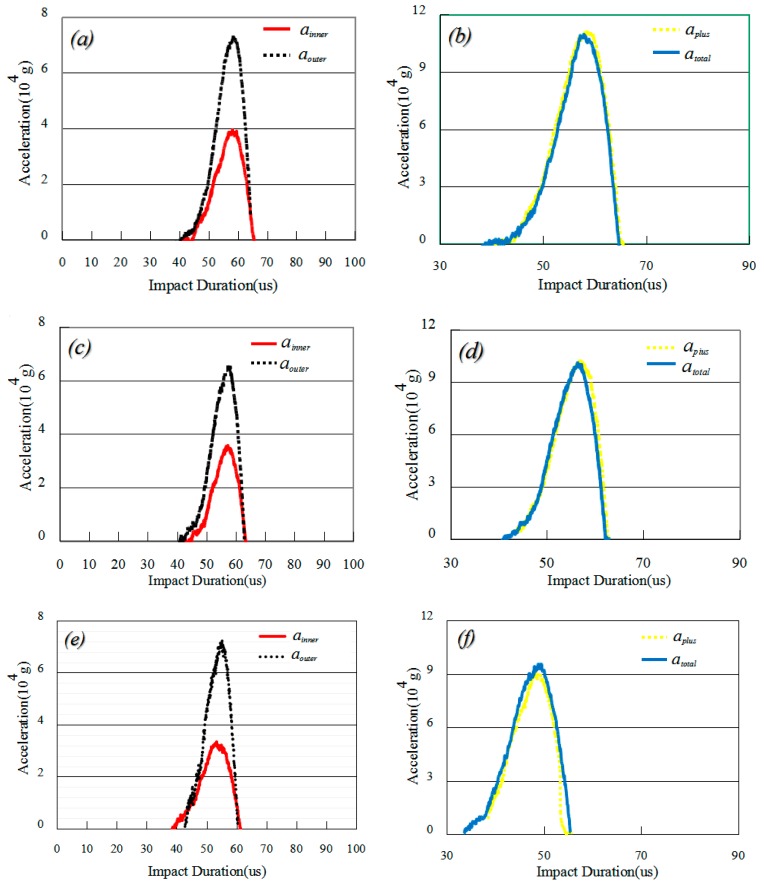
Experimentally determined accelerations under impacts from the inner (*a_inner_*, *solid lines*) and outer (*a_outer_*, *dotted lines*) bullets as functions of impact duration, as detected by the Doppler frequency shift of the mounted base. (**a**), (**c**), and (**e**) show the cases impacted by the bullets separately; (**b**), (**d**), and (**f**) show the cases impacted by both bullets simultaneously (*a_total_*, *solid lines*). The dotted lines in (**b**), (**d**), and (**f**) represent the sums of the accelerations (*a_plus_*) of the inner and outer bullets in (**a**), (**c**), and (**e**), respectively.

Figure 5 shows the experimentally determined accelerations under impact from the inner (*a_inner_*, *solid lines*) and outer (*a_outer_*, *dotted lines*) bullets as functions of the impact duration for the system without the accelerometer, as detected by the Doppler frequency shift of the mounted base. Here, Figure 5a,c,e show the cases impacted by bullets separately, and Figure 5b,d,f show the cases impacted by both bullets simultaneously (*a_total_*, *solid lines*). The dotted lines in Figure 5d, f represent the sums of the accelerations (*a_plus_*) of the inner and outer bullets in Figure 5a,c,e, respectively.

For the process of the inner bullet impact upon the Hopkinson bar, because of response delay, there are no accelerations (Doppler frequency shift) from 0 to 38 μs in Figure 5a, from 0 to 40 μs in Figure 5c, or from 0 to 42 μs in Figure 5e (*solid lines*) after the inner bullet impacts the bar. The accelerations then increase to the maximum with a value of 3.816 × 10^4^ *g* at 52 μs, 3.524 × 10^4^ *g* at 51 μs, and 3.240 × 10^4^ *g* at 52 μs in Figure 5a,c,e, respectively. Subsequently, the accelerations decrease to zero between 52 and 66 μs, 51 and 62 μs, and 52 and 62 μs in Figure 5a,c,e, respectively.

For the process of the outer bullet impact upon the Hopkinson bar, we have similar experimental results. There are no accelerations between 0 and 38 μs, 0 and 40 μs, or 0 and 42 μs after the outer bullet impacts the bar in Figure 5a,c,e (*dotted lines*), respectively. The accelerations then increase to the maximum, with values of 7.204 × 10^4^ *g* at 52 μs, 6.493 × 10^4^ *g* at 51 μs, and 6.595 × 10^4^ *g* at 52 μs in Figure 5a,c,e, respectively. Subsequently, the accelerations decrease to zero between 52 and 66 μs, 51 and 62 μs, and 52 and 62 μs in Figure 5a,c,e, respectively.

For the process of simultaneous inner and outer bullet impact upon the Hopkinson bar, the accelerations (*a_total_*) increase to the maximum values of 10.915 × 10^4^ *g* at 52 μs, 10.248 × 10^4^ *g* at 51 μs, and 9.436 × 10^4^ *g* of 52 μs in Figure 5b,d,f (*solid lines*), respectively. The other experimental tendencies and phenomena are the same as the inner and outer bullets impacting the bar in Figure 5a,c,e, respectively. In order to compare the experimental results, we sum the accelerations (*a_plus_*) as functions of the impact durations induced by the inner and outer bullets in Figure 5a,c,e. These results are shown by the dotted lines in Figure 5b,d,f. The peak accelerations of *a_inner_* and *a_outer_* in Figure 5a,c,e are also added together and summarized in Table 2. Comparing *a_total_* with *a_plus_* in Figure 5b,d,f and Table 2, we find that the accelerations, *a_plus_*, are well-consistent with those of *a_total_* for the process of impacting upon the Hopkinson bar using the inner and outer bullets at the same air pressure, simultaneously.

The dynamic linearities *σ*_1_, *σ*_2_, and *σ*_3_, were obtained from Equation (10) and are shown in Table 2. The systematic dynamic linearity, *σ_total_*, was calculated from the averaged *σ*_1_, *σ*_2_, and *σ*_3_ values as follows:

(17) $$\begin{array}{ll} \sigma_{total} & =\frac{\sigma_{1}+\sigma_{2}+\sigma_{3}}{3}\times 100\%\\ & =2.50\% \end{array}$$

From these experimental results, it is concluded that the dynamic linearity of the impact system is considered acceptable and that this system is of relative high reliability and accuracy.

### 4.3. Dynamic Linearity Results for a High*-g* Accelerometer

**Figure 6:**
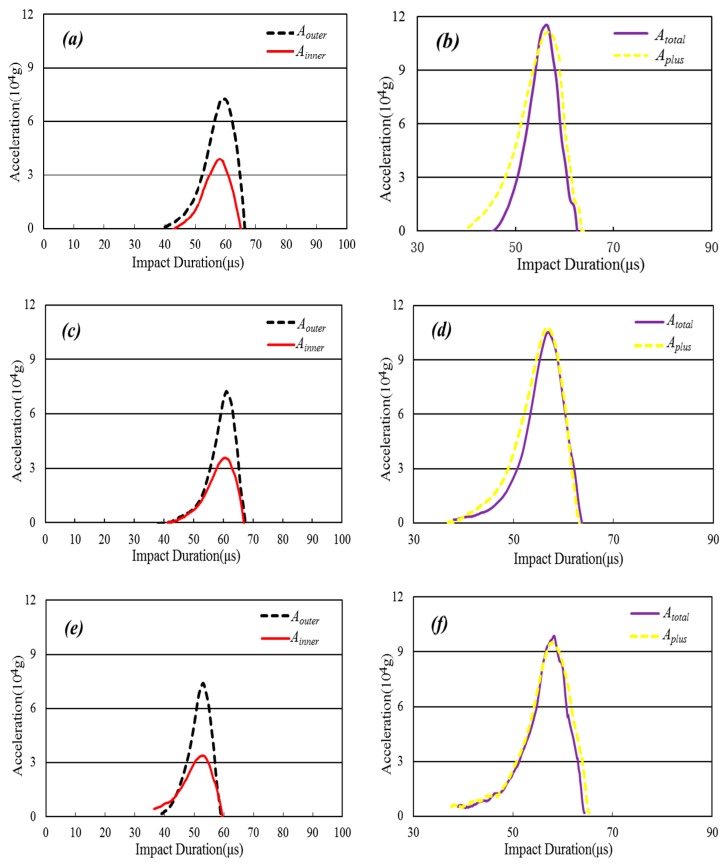
Accelerations as a function of the impact duration for the impact system, determined from the outputs of the accelerometers impacted by inner (*solid lines*) and outer bullets (*dotted lines*) ((**a**), (**c**), and (**e**)), and by both simultaneously (*solid line*) in (**b**), (**d**), and (**f**). The dotted lines in (**b**), (**d**), and (**f**) represent the sums of the accelerations of the inner and outer bullets in (**a**), (**c**), and (**e**), respectively.

In this experiment, the in-house manufactured piezoresistive accelerometer [30] with a measuring range of 1.5 × 10^5^ *g* and an anti-overload of 2.0 × 10^5^ *g* combined with the holder is mounted to the end side of a Hopkinson bar. Three pressures—0.160 MPa, 0.156 MPa, and 0.152 MPa—are applied to the dual-bullets. The outputs of the accelerometer dependence of the impact duration are recorded and shown in Figure 6a–f.

Figure 6 shows the experimental results of the inner (*A_inner_*) and outer (*A_outer_*) accelerations as functions of the impact duration for the accelerometer by detecting the output of the accelerometer impacted by the inner bullet (*solid lines*), outer bullet (*dotted lines*) (Figure 6a,c,e), and by both simultaneously (*A_total_*, *solid lines*) in Figure 6b,d,f. The dotted lines in Figure 6b,d,f represent the sums of the accelerations (*A_plus_*) of the inner and outer bullets in Figure 6a,c,e, respectively.

In Figure 6a, between 0 and 42 μs, the acceleration is almost zero. At approximately 42 μs, the acceleration rises, increasing to a maximum of 3.836 × 10^4^ *g* for the inner bullet and 7.256 × 10^4^ *g* for the outer bullet at 56 μs. From 56 to 68 μs, the acceleration decreases to zero. In Figure 6c, from 42 to 55 μs, the acceleration increases to a maximum of 3.462 × 10^4^ *g* for the inner bullet and 7.134 × 10^4^ *g* for the outer bullet. From 55 to 67 μs, the acceleration decreases to zero. In Figure 6e, from 40 to 51 μs, the acceleration increases to a maximum of 3.215 × 10^4^ *g* for the inner bullet and 6.753 × 10^4^ *g* for the outer one. From 51 to 62 μs, the acceleration decreases to zero.

In Figure 6b, from 0 to 42 μs, the acceleration is increased from zero to a maximum of 11.486 × 10^4^ *g* for a simultaneous impact by both bullets (*solid line*). From 56 to 68 μs, the acceleration decreases to almost zero. In Figure 6d, from 42 to 55 μs, the acceleration increases to a maximum of 10.226 × 10^4^ *g* under simultaneous impact. From 55 to 67 μs, the acceleration decreases to zero. In Figure 6f, from 40 to 51 μs, the acceleration increases to a maximum of 9.542 × 10^4^ *g* under the simultaneous impact. From 51 to 62 μs, the acceleration decreases to zero. 

To compare the experimental results, we sum the accelerations (*A_plus_*) as functions of the impact durations induced by the inner and outer bullets. The dotted lines in Figure 6b,d,f show these sums. The peaks of the accelerations of *A_inner_* and *A_outer_* in Figure 6a,c,e are also added together and summarized in Table 3. Comparing *A_total_* with *A_plus_* in Figure 6b,d,f and Table 3, we find that the accelerations *A_plus_* are well-consistent with *A_total_* for the process of impacting the Hopkinson bar using the inner and outer bullets at the same air pressure both simultaneously and separately.

The dynamic linearities of the accelerometer for each measurement—*σ’*_1_, *σ’*_2_, and *σ’*_3_—are obtained and shown in Table 3. The dynamic linearity *σ’_total_* of the accelerometer is calculated from the average of *σ’*_1_, *σ’*_2_, and *σ’*_3_, which is 3.88%.

From these results, it is concluded that the home-made accelerometers have high dynamic linearities. In particular, they can obtain higher accuracy of the acceleration than that of lower-dynamic linearity duration of a high*-g* impact. 

## 5. Conclusions

In this paper, a novel method for measuring the dynamic linearity of a high*-g* accelerometer using an impact experiment with a dual-warhead Hopkinson bar with laser interference was proposed. We theoretically determined that the dynamic linearity of the high*-g* accelerometer can be detected directly by comparing accelerations due to impacts by outer and inner bullets under the same condition, both separately and simultaneously. We then verified that the dynamic linearity of the impact system without the accelerometer is 2.5% experimentally, and the dynamic linearity of the high*-g* accelerometer was shown to be 3.88% after calibrating the system with the accelerometer. All of these results confirmed the reliability and feasibility of the dynamic linearity for the impact system and the home-made high*-g* accelerometers used in this method.

## Figures and Tables

**Table 1:** Parameters of the bullets.

Bullets	Inner Diameters (mm)	Outer Diameter (mm)	Length (mm)
Outer bullet	19.4	25.4	30
Inner bullet	—	11.2	30

**Table 2:** Peak accelerations of *a_inner_*, *a_outer_*, *a_plus_*, *a_total_*, and the dynamic linearities of the impact system.

	Test Results	*a_inner_* (10^4^ *g*)	*a_outer_* (10^4^ *g*)	*a_plus_* (10^4^ *g*)	*a_total_* (10^4^ *g*)	*σ_i_* (*i* = 1, 2, 3)
Test No.						
1		3.816	7.204	11.020	10.915	0.96%
2		3.524	6.493	10.017	10.248	2.31%
3		3.240	6.595	9.835	9.436	4.23%

**Table 3:** Peaks of the accelerations of *A_inner_*, *A_outer_*, *A_plus_*, and *A_total_*, and the dynamic linearity of the measured accelerometer.

	Test Results	*A_inner_* (10^4^ *g*)	*A_outer_* (10^4^ *g*)	*A_plus_* (10^4^ *g*)	*A_total_* (10^4^ *g*)	*σ’_I_* (*i* = 1, 2, 3)
Test No.						
1		3.836	7.256	11.092	11.486	3.55%
2		3.462	7.134	10.596	10.226	3.62%
3		3.215	6.753	9.968	9.542	4.46%
